# Human Relationships with Domestic and Other Animals: One Health, One Welfare, One Biology

**DOI:** 10.3390/ani10010043

**Published:** 2019-12-24

**Authors:** Ariel M Tarazona, Maria C Ceballos, Donald M Broom

**Affiliations:** 1 Facultad de Ciencias Agrarias, Departamento de Producción Animal Medellín, Universidad Nacional de Colombia, Antioquia 050034, Colombia; 2 Grupo ETCO, Group of Studies and Research in Animal Ethology and Ecology, Jaboticabal-SP 14884-900, Brazil; mceballos30@gmail.com; 3 Swine Teaching and Research Center, Department of Clinical Studies, New Bolton Center, School of Veterinary Medicine, University of Pennsylvania, Kennett Square, PA 19348, USA; 4 St Catharine’s College and Department of Veterinary Medicine, University of Cambridge, Madingley Road, Cambridge CB3 0ES, UK; dmb16@cam.ac.uk

**Keywords:** animal welfare, animal behavior, sentience, zoonoses, sustainability

## Abstract

**Simple Summary:**

In a situation where human actions are damaging much of the life of the world, it is important to remember that the basic concepts of biology, welfare, and health are the same for humans and all other animals. Human actions have wide consequences and we need to change the way we interact with other living beings. An understanding of the concepts of one health, one welfare, one biology, and their application to daily decisions about production systems, public policies, markets, and consumers could mitigate current negative impacts. In particular, an understanding of human relationships with animals used for food, work, or company helps in dealing with challenges concerning their use and system sustainability, including the animal’s welfare. Animal welfare should always be considered in our relationships with animals, not only for direct impacts, e.g., manipulations, but also for indirect effects, e.g., on the environment, disease spread, natural resource availability, culture, and society.

**Abstract:**

Excessive human population growth, uncontrolled use of natural resources, including deforestation, mining, wasteful systems, biodiversity reduction by agriculture, and damaging climate change affect the existence of all animals, including humans. This discussion is now urgent and people are rethinking their links with the animals we use for clothing, food, work, companionship, entertainment, and research. The concepts of one health, one welfare, and one biology are discussed as a background to driving global change. Nothing should be exploited without considering the ethics of the action and the consequences. This review concerns domesticated animals, including those used for human consumption of meat, eggs, and milk; horses kept for work; and dogs kept for company. Animal welfare includes health, emotional state, and comfort while moving and resting, and is affected by possibilities to show behavior and relationships with others of the same species or with humans. We show some examples of the relations between humans and domesticated animals in the environmental context, including zoonotic diseases, and consider the consequences and the new paradigms resulting from current awareness.

## 1. Introduction

### 1.1. Who Are We?

From a biological point of view, each human is an animal, a mammal, an ape with a scientific name like other animals *Homo sapiens* [1]. Many studies have shown that humans are different from other animals only in degree, not in terms of the general aspects of the biological functioning of their genome, body, or brain. There are differences in the way that human and other brains work, but humans share each of the brain systems with many other animals [2,3,4,5]. Whilst there are differences in the anatomical areas in which functional mechanisms occur, the actual functions that occur in humans also occur in other species. For example, the high-level cognitive functioning of birds like crows and parrots occurs in a different anatomical area from that in mammals, and pain analysis occurs in different areas in different groups of fish and in mammals. The human frontal and pre-frontal cortex have some more complex activities compared with other animals. Humans have better mathematical logic, perception of time, complex reasoning, analytical capacity, and prediction of events than most other species [6]. However, many non-human animals use their brains to make complex decisions, plan for the future, have concepts of objects that are not present, use tools, communicate, deceive, and show empathy [4]. Examples of empathy include a chimpanzee responding to another during childbirth, a dolphin lifting a human swimmer in difficulty or a sick dolphin, and a pet dog responding to another individual that is in pain; some of these are shown in [7,8,9]. The basic concepts of biology are the same for humans and other species and almost all biological systems occur in all vertebrates, including humans, so if each human is considered important, each other vertebrate individual could reasonably be considered to be important [10]. Contrary to the teachings of some religions, humans are animals; they have few differences from other animals and do not have to be considered as special in the sense of being more important [11]. The logical question raised here concerns how great a difference between one species and others requires that the species be valued more, or valued so much that other species are hardly valued at all. Humans can do much harm to other animals and to the local and world environment.

Thanks to advances in knowledge of biological phenomena related to reproduction, we know that animals with sexual reproduction (including humans) come from the DNA contained in an ovule (maternal) and a spermatozoon (paternal), producing the genotype that interacts with the environment to result in the phenotype of the new individual [12]. The probability of each individual existing is very low. Each human comes from one spermatozoon out of approximately 200 million [13], that merges with one of approximately 300 thousand potential ovules [14]. To this, we add the improbability that the parents of that individual existed, and also their grandparents, great grandparents, and so on, for generations. Every cow (*Bos taurus*, *B. indicus*), pig (*Sus scrofa*), dog (*Canis familiaris, C. lupus)*, cat (*Felis sylvestris catus*), rainbow trout (*Onchorhynchus mykiss)*, horse (*Equus caballus),* and human share this improbability in general terms. Being aware of the improbability of life, its short duration and its fragility, we could assign more value to and have more respect for other equally improbable life forms. When making use of animals for food, work, or companionship, we could consider that each is a unique and unrepeatable life.

During human evolution, there have been specialist developments, such as the thumb becoming opposed to the other fingers and the brain structure that allows grammar in language [15]. Other kinds of animals developed other specializations that are different adaptations from those of humans, not worse adaptations. Humans developed physical abilities to make things and a complex brain. Is this the ultimate that is possible? Future beings are likely to have greater abilities and current human abilities are already in some ways surpassed by the robots and other machines that are created by humans to replace us in many daily tasks [16]. A key adaptation in humans and other animals has been the evolution of feelings and emotions such as fear, anger, pleasure, and pain that facilitate learning and other components of environmental control systems [17,18]

Humans are more similar to other animals than most people think. The DNA of all vertebrates including humans has far more similarities across groups than differences [19]. The differences that some people present as exclusive to humans, such as language, emotions, the notion of culture or society, cooperation and altruism, have been reported in other beings, with scientific evidence demonstrating their existence in various groups of animals [4,10]. Indeed, some non-human species can be considered as moral agents [3,11]. In addition to our production of elaborate artistic outputs, two qualities that we might not share with non-human animals are that we produce much non-organic waste and we have the potential to decide more about our diet and environmental impact. An answer to the question “Who are we” is that we are the sentient beings living on earth.

As mentioned above, there is only one biology and all of the basic concepts, including welfare and health, have the same meaning for all animals. The concept of welfare as defined by Broom in 1986: “the welfare of an individual is its state as regards its attempts to cope with its environment” [20], includes feelings, health, and other mechanisms for coping. It clearly applies to humans and to any other living animal. Health is an important part of welfare and is the state of an individual, as regards its attempts to cope with pathology [21].

### 1.2. Where Are We?

Despite the wide-ranging effects of humans during 200,000 years and recent loudly-voiced campaigns for resource conservation, the reduction of waste, and the responsibility of each person for the care of the planet, little has actually been achieved to stop us from being one more species within the 6th great extinction [22,23]. In a universe 13,700 million years old, on a planet 4,500 million years old [24], our species is very young compared with jellyfish 500 million years old [25] or cockroaches 350 million [26]. Only in the last 13,000 years have humans created substantial settlements and utilised many plant and animal species [27,28]. Domestication of species such as goats, sheep, pigs, cows, dogs, and cats [4,29,30] and the evolution of human-animal relationships [31,32] have been described. We consider here how we have modulated our relationships with animals, negatively and positively and the impacts of the sciences of ethology and animal welfare. The history of animal welfare science is discussed by Broom [4,33].

Humans have long seen the world as a pantry, as a warehouse of materials that could be exploited and used at will, as if they were infinite and as if there were no consequences of material extraction or use [34]. Energy from coal, steam, oil, and oil derivatives led to humans generating much non-organic waste. This can remain unchanged for decades, centuries, or perhaps millennia, generating unpredictable consequences for the integrity of the matrix of life on the planet [35]. As a consequence of the human actions of the last two centuries, we are currently facing climate change [36], emerging diseases derived from animal population management [37], environmental pollution [38], deforestation [39], loss of ecosystems due to mining [40], and loss of biodiversity on cultivated land because of herbicide use, pesticide use, and other agricultural practices [41,42]. The changes in our relationship with domestic animals, associated with the intensification of farming, has resulted in many animal welfare problems. In the last few decades, the public perception of consumption has changed and has generated demands for the creation of laws, codes of practice, and public policies for the improvement of animal welfare in many countries [4,43]. Currently, animal welfare has been accepted as a key issue by FAO and OIE, is a public morality issue accepted by WTO, and is an integral part of the sustainability criteria of animal production systems [41,44]. 

### 1.3. Where Are We Going?

There is currently increasing production of meat, milk, and eggs, as demanded by the growing human population. Industrialized systems require a large number of resources such as water, soil, fertilizers, and fuels [45,46,47] and the use of world resources is often inefficient. They generate significant amounts of waste such as faeces, urine, materials used as bedding, and by-products not used in human food such as bones, leather, feathers, and hair. In addition to the potential effect of waste, water and air pollution, products may also contain residues of medications or antibiotics and this is a significant problem to solve, for example because of their effect on native aquatic populations [48]. Some of these systems have negative consequences for aspects of sustainability, including animal welfare. However, since good welfare generally results in higher production efficiency, we must use systems with good welfare that optimize the use of resources such as water and soil, use alternative energies such as solar or wind, and generate less and cleaner waste.

Although currently there are many initiatives to change traditional industrialized systems to cleaner alternatives that use world resources better, the reality is that the human demand for animal protein, working animals, and companion animals far exceeds the rate of transformation of those systems. Given this situation, we present some of the challenges concerning the welfare of domesticated animals. The concepts of one health and one welfare [49] are becoming widely accepted and we add “one biology”. The one biology concept implies that the biological principles are exactly the same for humans and all other animals, although there are specific differences between species and between individuals. Investigating biology means investigating humans together with all other species. Ecological, conservation, and other environmental principles should not be thought of in different ways in relation to humans and other species. Whilst different kinds of animals have different needs, the concept of health and the concept of welfare are exactly the same for a human, a pig, and an octopus (*Octopus vulgaris*). Consequently, decisions about relationships between humans, other animals, and the environment should respect all biological aspects of all living beings. No production system is isolated and devoid of impact on or consequences for the local and world environment. An interesting example of the relationships between disease, welfare, and socio-economic issues is presented by Thumbi et al. [50] This study, from sub-Saharan Africa, shows linkages between human and animal health, and the consequences of averting human disease for malnutrition, household educational attainment, and income levels [50]. New challenges arise regarding requirements for an animal production system, among them, the importance of the role of animal welfare in sustainability, the development of public policies and standards, precise methods of evaluation, and the importance of understanding animal welfare science in order to respond to these challenges [51].

## 2. Current Trends in Animal Production Systems

In the 1960s, a change in human perception of animal production systems involved considering inadequate animal housing and management in industrialized systems that cause pain and suffering to the animals. Publicity about this, and demands from the public and politicians for accurate information about the animals, unleashed a series of reactions that eventually led to the consolidation over time of animal welfare science. This provides knowledge concerning the needs of animals and scientific evidence about how well individuals are coping with their environment. Using this, we can provide each species of animal that we keep with the most appropriate housing and management conditions for their physical condition, mental state, emotional balance, and for appropriate expression of behaviors [52]. 

It has been recommended that animal welfare should be considered in relation to “quality of life” and “a life worth living” [53,54,55]. Quality of life means the same as welfare, although not normally used for short time periods, so can be measured [56]. The measures of welfare are objective. However, there is a difficulty with saying whether or not a life is worth living, as the decision about this involves subjective human judgement [4,54,57]. We consider that it is better to rely on objective measures such as health, body condition, physiological and behavioral measures of welfare, and understanding the needs of animals and the supply of resources. In order to be able to judge what are the key factors affecting the welfare of farm animals, we have to consider the systems used, some of their physical components, and interactions between humans and farm animals, as described in the following sections. Recent welfare research has increasingly focused, not just on negative effects but on positive welfare with conditions provided where negative experiences, such as isolation, pain, fear, stress, are minimized and the opportunities to have positive experiences, such as grooming, rest, play and affiliative behaviors, are provided [55,56,57,58,59,60]. The history and bases of animal welfare science have been addressed [33,61,62].

## 3. Production Systems and Animal Welfare

Industrialized high-density systems, for any of the farm species, have common characteristics associated with animal welfare problems, especially those associated with the supply of resources and management in relation to the needs of the animals [63]. Since the characteristics are specific to each species, age, breed, sex, size, etc., we list the characteristics and make a short description of the main problems in each species.

## 4. Welfare Problems Due to The Supply of Resources

The first resource is the place where animals live and the existing infrastructure. Facilities are essential because the animal interacts by direct contact with surfaces such as floors, walls, columns, and doors; this interaction can generate discomfort, lacerations, or increase the risk of behavioral problems or diseases. Insufficient space per animal is a very common problem for housed animals.

### 4.1. Housing Design

The quality of the floors of the facilities where the animals live for most of the time have a big effect on welfare, as do surfaces of paths where the animals move within the system, for example to milking, loading, unloading, crowding pen, or squeeze-chute. Ways in which characteristics such as material, nature of gaps in floors, drainage, roughness, slipperiness, and dirtiness can affect the welfare include: Physical damage to the animal’s feet, discomfort, inadequate rest postures, or difficulty in moving; discomfort during rest due to hardness of the floor, excessive wet or dirt; and increased risk of lameness disorders, respiratory infections, mastitis or endometritis due to dirtiness [64,65,66,67]. The materials of the walls may damage the animals, walls should be free of irregularities and elements that could potentially cause injury such as wire, ties, nails, screws, metal projections, or wood clips [68]. The animal may use the walls as a thermoregulation mechanism by heat exchange through direct contact so good building materials are essential for the maintenance of good welfare. The material of the roof can affect conduction of heat from the outside to the interior of the building [69] and alter the microenvironment in which animals live [70]. When not properly maintained, rodents or birds nesting under the roof are potential vectors of pathogenic microorganisms or parasites for both domestic animals and humans.

The building structures, and other components of the environment provided for domestic animals, interact with the weather in the region to produce microenvironment conditions for the animals [71]. Specialized equipment may generate or control wind, cold, heat, humidity, or other conditions. Ventilation is essential in buildings and vehicles for both thermoregulation and dissipation of aversive odors and harmful gases. Light is important because it allows many of the animals’ natural behaviors; for example, in mares and sows, it is crucial for reproduction [72]. Temperature control is vital for homeostasis, and its absence can lead to thermal stress and sometimes parasite and disease proliferation. A small decrease in temperature can favor respiratory diseases, especially in juvenile animals. Thermal sensation is a consequence of temperature and humidity acting together. This perception by the animals is vital to avoid stress from both hyperthermia and hypothermia. Excessive air moisture decreases the rate of sweating and makes it difficult to exchange heat with the environment, and may cause heat shock in animals [73]. Solar radiation is essential in systems where animals live outdoors, not only in extensive systems but also in intensive confinement systems without shade. Excessive radiation in non-adapted animals can cause skin burns and has been associated with some types of dermal carcinomas, especially in depigmented animals [74]. The lack of shade can also cause heat stress [75].

### 4.2. Food and Water 

Various studies in several animal species have shown that many characteristics of feeders and drinkers, such as material, size, height, and distribution, are important for animals. Inadequate designs and insufficient numbers of feeders and drinkers can generate serious welfare problems due to agonistic social behaviors or simply because they do not allow some individuals to access food and water resources [58]. Additionally, feeders with inadequate preventive maintenance can become pathogen sources. Damaged feeders and drinking troughs can increase the risk of injury to animals. 

Food is a valuable resource for an animal that must meet three fundamental characteristics to avoid causing poor welfare. It must be provided in appropriate quantity, in good quality, and be accessible to all animals in a group. Therefore, factors such as the form of presentation of the food, the smell, color, taste, texture, and arrangement in space can determine whether or not all animals can access the resource when they have the motivation to do so [58]. Domestic species are willing to work hard to access water. Drinkers must be designed according to how animals show their water consumption behavior and be adapted to their anatomical structure. The water must be of sufficient quality to prevent problems in animals and be free of disturbing odors and tastes. The mishandling of both water and drinkers can generate many welfare problems due to dehydration; especially when the animal is under heat stress and its need for water increased. Any direct water consumption from natural water sources should take account of ecological implications. The amount of water used in beef production is described for four systems by Broom [47]

### 4.3. Bedding and Objects in Animal Accommodation

In many animal production systems, various materials are used as bedding in a building that houses animals. Among these materials are sawdust or wood chips, straw, synthetic materials, and agro-industry by-products such as rice hulls. The materials used in construction or bedding can also generate welfare problems, on the one hand for animal safety and on the other as a potential vector of diseases or parasites. Small particles forming dust can cause respiratory problems, for humans [76] but also for animals such as pigs and calves [77].

Objects in farm animal living accommodation can provide environmental enrichment to improve the captive environment and may prevent or reduce abnormal behaviors [78]. Objects can be fixed, mobile on the ground, or suspended, and designed to be touched, smelled, bitten or pushed. Objects within a system must be made of an innocuous material that does not injure the animals. Pigs prefer deformable and manipulable materials. Wooden objects can be dangerous when they break, leaving splinters that the animal can swallow. Plastic bags can cause asphyxiation and metal objects can cause cuts so all objects should be appropriate for the species.

## 5. Welfare Problems Due to Animal Grouping and Handling

How humans interact with animals can lead to animal welfare problems [79]. This is a subject for the training of staff. All those who handle animals should receive training. The way in which animals are distributed in limited and enclosed areas should be appropriate for their needs. Almost all farm animals are highly gregarious [80], and in the wild, they move in groups of defined numbers within a territorial or home range space [81]. The grouping of animals by human, on many occasions without taking account of their biology or former group composition, can lead to the expression of agonistic behaviors and serious welfare problems due to injuries and stress. The biology of the species must be taken into account, to make an adequate grouping, considering: the number of animals per group, sex, and physiological status [82]. An adequate density should allow freedom of movement of the animals and easy changes of posture. There should be space, at any given time, for all animals to lie down simultaneously with sufficient space between them for thermoregulation and movement. The group size should allow the recognition of all individuals and stable social cohesion [83].

The movement of animals from one place to another should be done in such a way that it does not generate stress or injuries in animals [84]. Driving methods should avoid direct contact, including blows, use of sticks, ropes, electric goads, or sharp objects. It should also be done in a quiet environment and at a steady pace, without causing fear to the animals. There should be avoidance of shouting and high-pitched sounds, since scared animals have a higher risk of slipping, falling, and injury [85,86]. Tools to facilitate driving without generating pain or stress in animals include flags and boards. The design and structure of buildings and races play an essential role in allowing easy movement of animals and ensuring good welfare. Poorly designed and poorly managed buildings lead to many welfare problems [85]. The training of stockpeople has been shown to substantially improve animal handling on farms, because it helps them understand the behavior of farm animals, positively modifies their attitudes, and improves their conduct toward animals [87,88,89].

A poor method for capturing animals can cause the animal to collide with hard structures and become injured [88]. Once captured, the animal can be immobilized, if necessary. This immobilization requires adequate equipment, specialized races and other structures, established protocols, and experienced handling by the person handling the animal [89]. Poor quality capture and immobilization generates risks for both the animal and the person who is handling it. 

### Pain Management

In animal production systems, many procedures generate pain in animals (see Table 1); both acute and chronic pain have negative consequences for animal welfare [90]. Pain is possibly one of the consequences with the most negative perception for the general public, so improvement in pain management is essential for the animal production industry. Pain can be managed basically in three ways: abolishing painful practice, using anesthesia, and using analgesia. Some practices, such as tail-docking may be abolished because there is no evidence that they generate a benefit for production in good conditions, for quality of milk, or for animal health. The question to consider is whether or not the practice executed is essential and justifiable from various points of view, including animal welfare [59]. Another example is surgical castration, which has been demonstrated to be painful by much research [91,92,93]. When partial strategies to reduce the pain caused by surgical castration are used, just analgesia or just anesthesia, pain is not completely prevented [94,95]. The pain induced during castration, and during wound healing, leads to activation of adrenal and sympathetic axes [96]. It is well known that adrenal hormones affect immune function, reducing NK cell activity, lymphocyte population, lymphocyte proliferation, antibody production, and reactivation of latent viral infections [97]. These effects have severe consequences for health, including delayed wound healing and impaired responses to vaccination [96]. When a painful practice generates a high level of pain, local or general anesthesia should be used. When a procedure generates pain that is known to remain for a substantial time after the end of the intervention, it is necessary to use analgesia in addition to anesthesia. This pain alleviation is important because some practices in farm animals are legally accepted, for either tradition, cost, convenience, veterinary treatment, sport, or breeding reasons [62].

## 6. The Main Welfare Challenges in Production Systems

Where stress means an environmental effect on an individual which over taxes its control systems and results in adverse consequences and eventually reduced fitness [96], some welfare problems are associated with stress. Some involve pain, some are long-term, and others are short-term. Table 1 is a summary of the most common welfare problems in farm animals. In addition to these problems for the animals, there can be difficulties for care staff if they receive different messages about animal care from owners, veterinarians, and colleagues.

The “one welfare” concept makes it clear that human welfare and non-human animal welfare mean the same thing, and that poor welfare often leads to poor health and other poor welfare, sometimes because the poor welfare suppresses immune system function. Poor welfare that makes production systems more inefficient is likely to have negative effects on human welfare too. Both stress and pain require energy for compensation, so part of the energy consumed by the animal is used to try to deal with welfare problems. The additional energy expenditure resulting from poor welfare reduces productive efficiency and has consequences for the sustainability of the system.

There is already scientific information about many of the causes of poor welfare and practical information about how to improve welfare, yet preventable mistakes that lead to many economic losses are made [98]. Some welfare problems mentioned in Table 1 require that genetic selection of farm animals be modified to reduce productivity since the animals are metabolically over-taxed. There is a trade-off here between what is best for welfare and the desirability of use of efficient production systems so that environmental impact is reduced [42,47].

## 7. Animal Production Systems, Human Health, and Environmental Impact

Under the concept of “one health”, it is understood that the health of humans and other animals are the same concept and area of effect [154]; therefore, the maintenance of health in production systems has positive repercussions on the assurance of good human health and mean health in all populations. The concept of One Welfare arises as a complement to the one health approach [154,155]; The fields of human and non-human welfare are empowered by addressing more effectively the connection between policy and science, including environmental science and sustainability. There is interconnection between the environment and the welfare of all animals, including humans [156]. Since the most prolonged and important human interaction with other animals is with pets and indirectly with the products of farm animals, Table 2 shows the main zoonotic diseases, excluding many that are transmitted by wild animals.

## 8. Consequences of Human-Animal Relations for The Environment

In addition to the human-animal interaction problem of humans getting diseases directly from non-human animals, a further major problem is that human actions are having adverse effects on ecosystems and the whole world environment. This paradigm is not new; it began last century with the issue of climate change and global warming, which triggered alarms, especially for considering animal production systems as significantly responsible for greenhouse gas production and having a very large water and carbon footprint [157]. The need for a focus of attention on the impact of animal production on the environment has been strongly emphasized [158]. Livestock are described as one of the biggest factors responsible for climate change. Whilst there is now evidence that refutes several of these claims [159], the publication generated such a degree of discomfort with its assertions that it encouraged scientific investigation of the subject and efforts to refute the conclusions [159,160,161,162]. One of the issues is that data often refer to only one system and this can be misleading. For example, whilst [158] and many other publications use largely beef feedlot system data when referring to beef impacts, beef production from feedlot systems is much worse for conserved water usage than extensive pasture, fertilized pasture, and semi-intensive silvopastoral systems; the last is a form of agroforestry, typically integrating three-level plant production, including improved pastures, high densities fodder shrubs with edible leaves, and timber, fruit or palm trees, that may also have edible leaves [163]. Land use is highest for extensive pasture and higher for feedlot systems than that for semi-intensive silvopastoral systems [47]. Another important issue is that herbivorous animals such as cattle, sheep and some farmed fish can consume material that humans cannot consume. If the food products of these herbivorous animals are consumed, world resources are used more efficiently if they are not fed grain or other potential human food. It can be considered wasteful for human food to be fed to animals with a big loss of food availability to humans [163,164]. Broom [165] states that any effect which the general public find unacceptable makes a system unsustainable, for example: inefficient use of world resources, adverse effects on human health, negative impacts on animal welfare, harmful environmental effects, unacceptable genetic modification, not being “fair trade” or damage to rural communities. Some alternative solutions are agroforestry and the use of silvopastoral systems [166,167]. It has been shown that applying the principles of animal welfare for health assurance and maintenance of productive efficiency also has a direct impact on sustainability [163] and that some systems with high animal welfare standards are good for most other aspects of sustainability [168].

As an example of this are the set of “sustainable development goals (SDGs)” that the United Nations adopted in 2015, to reach 2030, a scenario without hunger and poverty, safe from the critical effects of climate change and loss of biodiversity [169]. A workshop called “Animal Welfare and the Sustainable Development Goals” in 2018 at the Swedish University of Agricultural Science had a group of 12 active participants, from eight countries, with an academic background in agricultural or veterinary science. They evaluated every goal and found that although animal welfare was not explicitly mentioned in the SDGs, working to achieve those goals is compatible with working the improvement of animal welfare [170]. 

## 9. Conclusions

Consideration of the welfare, including the health, of humans and consideration of welfare and disease spread in other animals, cannot be separated from evaluating the consequences of human decisions about the natural world. We ought to try to take account of each living being, and each community, population, and ecosystem; otherwise, human as well as other life could be endangered. Proposals are made to take account of the concepts of one health, one welfare, one biology; and to apply them to daily decisions about production systems. Animal welfare is fundamental to making decisions about the global consequences of our relationships with domestic animals, not only the direct impact of manipulating them, but also the effect on the environment, on disease spread, natural resource availability, culture, and society. A series of possible solutions are presented for a wide range of animal welfare problems, taking account, where relevant, of zoonotic and other diseases.

## Figures and Tables

**Table 1:** Widespread welfare issues in domestic animals with usual causes and possible solutions.

Species	Welfare Consequences	From Genetic Selection and Resources	From Animal Handling	Solutions	References
Dairy cattle	Mastitis	Metabolic pressure from high milk yield Dirty infrastructure	Bad milking practices	Select and feed for lower yield Prevention with cleaning and disinfection Application of good milking practices	[99,100]
	Lameness	Metabolic pressure from high milk yield Floor quality (hardness, wet, dirt) Food	Absence of podiatry	Select and feed for lower yield Improve floor condition Preventive podiatry Nutritional management	[101]
	Metritis and other reproductive disorders	Metabolic pressure from high milk yield Dirty and wet floor Food inadequacy	Bad peripartum protocols	Select and feed for lower yield Cleaning and disinfection of surfaces Protocols of good management peripartum Nutritional management	[102]
	Heat stress	Absence of overheating prevention mechanisms Absence of heat dissipation mechanisms Food	High density Poor selection of breeds to put on site	Shade Fans Water sprinklers Reduce density Use adapted breeds	[103]
	Diarrhoea	Dirty water Mishandling of colostrum	Bad practices of grouping animals	Good calf management practices Improve water quality Good colostrum management	[104,105]
	Respiratory diseases	Dusty buildings Cold High humidity	Bad practices of grouping animals	Dry, clean and larger building Cold protection Vaccination	[106]
	Pain from surgical interventions	Absence of analgesia, anaesthesia	Dehorning disbudding Tail docking Bad restraining practices	Abolish unnecessary procedures Use anaesthesia and analgesia Improve restrained protocols	[107]
	Social stress and abnormal behaviours (fights, stereotypies etc.)	Insufficient space and needs not met Scarce or inaccessible resources for all animals (includes area, food, water, enrichments)	Bad practices of grouping, regrouping and density management	Avoid individual housing in small pens. Good grouping practices Ensure that resources are available for all animals	[58]
Beef cattle	Lameness Swollen joints Arthritis	Genetic selection for fast growth Floor quality (Hardness, wet, dirt)	Absence of podiatry Poor herd handling	Avoid fastest growing strains Improve floor condition Preventive podiatry Nutritional management Improve management protocols	[108,109,110]
	Heat stress	Absence of overheating prevention mechanisms Absence of heat dissipation mechanisms Inappropriate food	Absence of shadow Very heavy animals Breeds with dark hair	Provide shade Fans Water sprinklers Reduce density Adapted breeds	[75,111,112]
	Underfeeding	Poor supply of forage in extensive systems	Poor herd handling	Improve grazing system Silvopastoral systems	[113]
	Pain from surgical interventions	Absence of analgesia, anaesthesia	Dehorning Disbudding Bad restrained practices Castration Hot-iron branding	Abolish unnecessary procedures Use anaesthesia and analgesia Improve restraining protocols	[107,114,115]
	Social stress and abnormal behaviours (fights)	Scarce or inaccessible resources for all animals (includes area, food, water, enrichments)	Bad practices of grouping, regrouping and density management	Good grouping practices Ensure that resources are available for all animals	[83]
Pigs	Neonatal mortality	Infrastructure	Bad peripartum practices	Reduce sow stress before and during parturition Improve facilities	[116,117]
	Weaning	Food change	Abrupt separation from the mother at an early age Mix of unknown groups	Make groups with established hierarchy from the beginning Do not mix groups Leave the animals more days with the mother	[118]
	Social stress and abnormal behaviours (bites, redirected behaviour and stereotypes)	Individual confinement Scarce or inaccessible resources for all animals (includes area, food, water, environmental enrichments)	Bad practices of grouping, regrouping and density management	Do not confine in stall or tether Good grouping practices Ensure that resources are available for all animals	[58,119]
	Heat stress	Absence of overheating prevention mechanisms Absence of heat dissipation mechanisms Food	High density Poor selection of breeds to put on site	Fans Water sprinklers Reduce density Adapted breeds	[120]
	Bursitis	Inappropriate genetic selection Infrastructure	Bad grouping practices	Genetic selection to minimise Infrastructure improvement	[121]
	Lameness	Floor quality (hardness, wet, dirt) Pen conditions Space restriction	Lack of preventive actions	Improve floor condition Preventive podiatry	[122]
	Pain from surgical interventions	Absence of analgesia, anaesthesia	Tail-docking Teeth resection Castration Ear-tagging Notching Bad restrained practices	Abolish unnecessary procedures Use anaesthesia and analgesia Improve restrained protocols Use immunocastration	[95,96,123]
Laying hens	Foot problems (dermatitis, bumble foot, hyperketosis, excessive claw growth)	Floor and facilities quality (hardness, wet, dirt)	Lack of preventive actions	Litter hygiene Use low pressure-loading perches Provide scratching surfaces Food Choose healthy breed	[124]
	Injurious behaviour (aggression feather pecking)	Scarce or not accessible resources for all animals (includes area, food, water, enrichment, perch to rest)	Housing density	Avoid conventional cages. Stable groups Ensure that resources are sufficient and available for all animals	[125,126,127]
	Osteoporosis (due to selection for high egg yield), keel bone fractures	Insufficient space for exercise. Inadequate diet	No evidence from animal handling	Provide space to exercise and meet all needs. Perch height/design Low pressure-loading perches Provide trajectory clear of objects for movement between perch & ground Breed for bone strength	[128,129]
	Mutilations	Absence of analgesia, anaesthesia	Beak-trimming Toe-clipping Dubbing De-spurring	Abolish unnecessary procedures. Use anaesthesia and analgesia	[125]
Meat poultry	Locomotion problems (poor walking ability, lameness) Dermatitis	Growth too fast for leg strength. Floor and facilities quality (hardness, wet, dirt)	Lack of preventive actions, e.g., feed less if growth too fast	Genetic selection for slower growth Reduce feed Improve litter hygiene	[130,131]
	Physiological disorders (ascities, sudden death syndrome)	Growth too fast for metabolic function	Lack of preventive actions, e.g., feed less if growth too fast	Genetic selection for slower growth Reduce feed	[132]
	Heat stress	Absence of overheating prevention mechanisms Absence of heat dissipation mechanisms Food	High density. Poor selection of breeds to put on site	Fans Reduce density Adapted breeds	[133]
Fish	Physiological disorders	Poor water quality	Stocking density too high	Ensure good aeration and water quality Appropriate and sufficient space Reduce stocking density	[134,135]
	Fin erosion	Infrastructure (floating cages, ponds)	Stocking density too high	Infrastructure preventive maintenance Reduce stocking density	[136]
	Hunger	Insufficient food	Fasting Poor food distribution	Reduce stocking density Improve food distribution so all fish get sufficient food	[137,138]
	Exposure to air	Insufficient space	Poor handling procedures	Avoid air exposure	[139]
	Aggression	Lack of resources Insufficient space	Stocking density too high	Reduce stocking density Good grouping practices Ensure that resources are available for all animals	[137]
Horses	Aversive Taming	Inadequate taming space	Soring and abusive training methods	Good taming and training practices	[140]
	Overload and overexertion (Carriage and by stockpeople)	Inappropriate saddles and bridles	Poor handling procedures	Appropriate calculation of load capacity Adequate design and use of saddles and bridles Improve handling procedures Appropriate workload timing	[140,141,142]
	Discomfort from uncomfortable saddles and bridles	Inappropriate saddles and bridles	Poor handling procedures	Adequate design and use of saddles and bridles Improve handling procedures	[143]
	Colic	Multifactorial	Poor handling procedures	Ensure water quality and availability Allow pasture access Avoid feeding hay on the ground in sandy areas Feed grain and pelleted feeds only when necessary Allow exercise Control parasites Ensure dental care	[144]
	Abnormal behaviour (including stereotypies)	Individual housing. Density. Absence of enrichments	Social isolation. Poor human-animal relationship	Avoid individual housing Allow socialisation Environmental enrichment Training	[58,145]
	Locomotion problems (lameness)	Floor and facility quality (Hardness, wet, dirt) Inadequate feeding Injurie	Lack of preventive actions	Improve floor condition Preventive podiatry	[141,145,146]
	Hunger and dehydration (working horses)	Absence of sufficient water. Absence of sufficient food	Bad practices in food handling	Adequate supply of food and water in quality, quantity, and availability	[141,142,145]
Dogs	Hunger and dehydration (stray dog)	Absence of sufficient water. Absence of sufficient food	Abandonment of animals Animals without owner	Control programs for stray animals Feeding in shelter Provide water and food supply	[147]
	Breed related conditions	Genetic selection for flat faces and other characters	Individuals allow breeding of dogs with genetic abnormalities	Avoid selection pressure for unadaptive “aesthetic” qualities Increase genetic diversity Do not allow dogs with flat faces to breed	[148]
	Obesity (pet dogs)	Overfeeding	Lack of exercise	Proper management of balanced diets Regular exercise	[149]
	Chronic pain (stray and pet dogs)	Failure to provide analgesia	Failure to use analgesia. Absence of veterinary treatment	Preventive veterinary medicine and timely pain treatment Use analgesia	[150]
	Behavioural problems (pet dogs)	Absence of enrichment Space	Absence of early education Inappropriate grouping Social isolation Poor human-animal relationship	Owner education Animal training Appropriate stimuli, do not leave dogs without other dogs or people for long periods Exercise Socialization	[151]
	Injuries (dog fighting)	Insufficient space	Inadequate grouping	Abolition of dogfights	[152]
	Mutilations	Absence of use of anaesthesia and/or analgesia	Ear cropping and tail docking	Abolish unnecessary procedures	[153]

**Table 2:** Most significant zoonotic diseases around the world. Listed by alphabetic order, not importance order. Adapted from the OIE-Listed diseases 2019.

Zoonotic Disease	Organism	Main Reservoirs
Animal influenza	Influenza A viruses	Pigs, poultry, humans
Anthrax	*Bacillus anthracis*	Livestock, environment, wild animals
Avian influenza	Influenza A viruses	Poultry, ducks
Bovine tuberculosis	*Mycobacterium bovis*	Cattle
Brucellosis	*Brucella* spp.	Cattle, goats, sheep, pigs
Campylobacteriosis	*Campylobacter* spp.	Poultry, other farm animals
Crimean-Congo haemorrhagic fever (CCHF)	CCHF virus	Livestock, ticks
Cryptosporidiosis	*Cryptosporidium* spp.	Cattle, sheep, pets
Cysticercosis/Taeniasis	*Taenia* spp.	Cattle, pigs
Erysipeloid	*Erysipelothrix rhusiopathiae*	Pigs, fish, environment
Fish tank/swimming pool granuloma	*Mycobacterium marinum*	Fish
Glanders	*Burkholderia mallei*	Horse, donkey, mule
Haemorrhagic colitis and haemolytic uraemic syndrome (HUS)	Shiga toxin-producing E. coli	Ruminants
Hendra virus infection	Hendra virus	Horses, bats
Hepatitis E	Hepatitis E virus	Pigs, wild boar, deer
Hydatid disease	*Echinococcus granulosus*	Dogs, sheep
Leptospirosis	*Leptospira* spp.	Ruminants
Listeriosis	*Listeria* spp.	Cattle, sheep
Louping ill	Louping ill virus	Sheep, grouse
Lyme disease	*Borrelia burgdorferi*	Sheep, ticks, rodents, deer, small mammals
Lymphocytic choriomeningitis	Lymphocytic choriomeningitis virus (LCMV)	Rodents
Orf	Orf virus	Sheep, goats
Ovine chlamydiosis	*Chlamydia abortus*	Sheep, farm animals
Pasteurellosis	*Pasteurella* spp.	Dogs, cats, many mammals
Psittacosis	*Chlamydia psittaci*	Psittacine birds, poultry, ducks
Q fever	*Coxiella burnetii*	Cattle, sheep, goats, cats
Rabies	Rabies virus and other lyssaviruses	Cattle, horses, dogs, foxes, haematophagous bats, cats
Rat bite fever	*Streptobacillus moniliformis*	Rats
Rift Valley fever	Rift Valley fever virus	Cattle, goats, sheep
Ringworm	Dermatophyte fungi	Many animal species
Salmonellosis	*Salmonella* spp.	Poultry, farm animals
Streptococcal sepsis	*Streptococcus suis*	Pigs
Streptococcal sepsis	*Streptococcus zooepidemicus*	Horses
Tickborne encephalitis	Tickborne encephalitis virus	Rodents, small mammals, livestock
Toxocariasis	*Toxocara canis/catis*	Dogs, cats
Toxoplasmosis	*Toxoplasma gondii*	Cats, ruminants
Trichinellosis	*Trichinella spiralis*	Pigs, wild boar
Zoonotic diphtheria	*Corynebacterium ulcerans*	Cattle, farm animals, dogs
